# Investigation of secretoneurin as a potential biomarker of brain injury in very preterm infants: A pilot study

**DOI:** 10.1371/journal.pone.0284096

**Published:** 2023-04-06

**Authors:** Anna Posod, Karina Wechselberger, Yasmin Pellkofer, Marlene Hammerl, Martina Urbanek, Eva Huber, Ursula Kiechl-Kohlendorfer, Elke Griesmaier

**Affiliations:** Department of Pediatrics II (Neonatology), Medical University of Innsbruck, Innsbruck, Tyrol, Austria; University of Modena and Reggio Emilia, ITALY

## Abstract

Neurodevelopmental impairment is a significant complication among survivors of preterm birth. To improve outcomes, reliable biomarkers for early detection of brain injury and prognostic assessment are required. Secretoneurin is a promising early biomarker of brain injury in adults and full-term neonates suffering from perinatal asphyxia. Data on preterm infants is currently lacking. The aim of this pilot study was to determine secretoneurin concentrations in preterm infants in the neonatal period, and to assess secretoneurin’s potential as a biomarker of preterm brain injury. We included 38 very preterm infants (VPI) born at <32 weeks’ gestation in the study. Secretoneurin concentrations were measured in serum samples obtained from the umbilical cord, at 48 hours and 3 weeks of life. Outcome measures included repeated cerebral ultrasonography, magnetic resonance imaging at term-equivalent age, general movements assessment, and neurodevelopmental assessment at a corrected age of 2 years by the Bayley Scales of Infant and Toddler Development, third edition (Bayley-III). In comparison to a term-born reference population, VPI had lower secretoneurin serum concentrations in umbilical cord blood and blood collected at 48 hours of life. When measured at 3 weeks of life, concentrations correlated with gestational age at birth. Secretoneurin concentrations did not differ between VPI with an imaging-based diagnosis of brain injury and those without, but when measured in umbilical cord blood and at 3 weeks of life correlated with and were predictive of Bayley-III motor and cognitive scale scores. Secretoneurin levels in VPI differ from term-born neonates. Secretoneurin seems unsuitable as a diagnostic biomarker of preterm brain injury, but bears some prognostic potential and is worthy of further investigation as a blood-based biomarker of preterm brain injury.

## Introduction

Preterm birth accounts for about 10% of all live births worldwide [1]. Improvements in perinatal care have markedly increased survival rates of preterm infants, but morbidity remains substantial and survivors continue to have high rates of neurodevelopmental impairment [2–5]. The aetiology of preterm brain injury is incompletely understood, but is considered multifactorial [6, 7]. Treatment focuses on best supportive care, as to date causal therapies are not available. Numerous promising treatment strategies are subject to investigations, and some may soon find their way into clinical trials [7–9]. A key challenge regarding treatment options is to identify infants who will most likely suffer from brain injury and neurodevelopmental impairment and, thus will benefit the most from timely therapeutic interventions. In recent years, several studies have evaluated the use of blood-based biomarkers to diagnose preterm brain injury and to predict neurodevelopmental outcome [10]. Secretoneurin, a highly conserved 33‐amino acid polypeptide, is a promising early biomarker of brain injury and unfavourable neurological outcome in adult patients following cardiopulmonary resuscitation [11]. Secretoneurin levels are also increased in adults diagnosed with ischaemic stroke [12] and in term neonates suffering from perinatal asphyxia with hypoxic-ischaemic encephalopathy [13]. Data on secretoneurin levels in preterm infants are currently lacking. To narrow this knowledge gap and to further evaluate the potential of secretoneurin as a biomarker of neonatal brain injury, we conducted a pilot study in the particularly vulnerable population of very preterm infants born at less than 32 weeks’ gestation.

The aim of the study was to investigate secretoneurin concentrations in very preterm infants as such, and to assess secretoneurin’s potential as a diagnostic and prognostic biomarker of preterm brain injury. Firstly, we determined secretoneurin concentrations in preterm-born infants at different time points in the neonatal period and put them in reference with available data from term-born neonates [13]. Based on the knowledge that secretoneurin is upregulated by hypoxia and is involved in inflammatory processes [14]–factors which play crucial roles in the injury cascade of neonatal brain injury [15]–we hypothesized that secretoneurin serum concentrations are elevated in preterm infants suffering from brain injury. To explore this hypothesis, we secondly compared secretoneurin concentrations in preterm infants with and without this diagnosis on imaging modalities. Thirdly, we evaluated the relationship between secretoneurin concentrations determined at different time points and neurodevelopmental outcome at a corrected age of 2 years.

## Materials and methods

### Statement of ethics

The study protocol was reviewed and approved by the local institutional review board (Ethics Committee of the Medical University of Innsbruck; approval number AN-2017-0046 371/4.1). The clinical trial was registered before patient enrolment (https://ctc.tirol-kliniken.at). Written informed consent to participate in the study was obtained from all participants’ parents or legal guardians in compliance with the Declaration of Helsinki.

### Study population and study design

The study was conducted as a prospective observational single centre study. Preterm infants born at less than 32 gestational weeks between June 2017 and November 2018 at our institution were considered eligible for the study. Written informed consent was obtained from legal guardians within 48 hours after birth. Exclusion criteria included major congenital anomalies and failure to obtain informed parental consent. Details on data collection are provided in the S1 File.

### Blood sampling

Blood samples were drawn in the context of routine blood collections. Blood samples were obtained from the umbilical cord (umbilical cord blood, UCB) and from study subjects at 48 hours of life as well as 3 weeks after birth.

### Secretoneurin radioimmunoassay

Collected blood samples were centrifuged at 3000 rpm for 10 minutes at 4°C. Sera were collected and stored at -80°C until further analysis. Secretoneurin concentrations were evaluated by a specific radioimmunoassay as described previously (detection limit 1–4 fmol/mL, inter- and intra-assay coefficients of variability <10%) [13, 16]. Reference values for secretoneurin serum concentrations in UCB and 48 hours of life were obtained from previously published data for healthy term neonates [13].

### Diagnostics of preterm brain injury

Infants received their first cerebral ultrasound during the first 72 hours of life and then underwent ultrasonographic imaging at least biweekly until discharge. Ultrasound investigations were performed and interpreted according to current recommendations by trained paediatricians/paediatric radiologists. Cerebral ultrasound pathology was defined as any abnormality observed at any examination time point. Germinal matrix and intraventricular haemorrhage (GMH-IVH) was graded according to Volpe [17, 18]. In addition to ultrasound, cerebral magnetic resonance imaging (MRI) was obtained at term-equivalent age [19]. Images were acquired at the local Department of Neuroradiology with a Siemens 3.0 Tesla scanner. Cerebral injury was graded as no, mild or severe injury [20]. Two operators blinded to clinical data evaluated all images. Consensus was reached upon discussion. General movements assessment was conducted at 52 weeks postmenstrual age by a standardized procedure [21]. Scoring was performed by at least two certified investigators.

Neurodevelopmental outcome was assessed by experienced psychologists at a corrected age of 2 years by the Bayley Scales of Infant and Toddler Development, third edition (Bayley-III) ([22], see also S1 File). Abnormal neurodevelopmental outcome was defined as a score of <85 comprising delay (score 70–84) and impairment (score <70) in any of the three domains cognitive scale, language scale, and/or motor scale.

### Statistical analysis

Statistical analyses were performed using IBM SPSS Statistics for Windows version 26 (IBM Corp., Armonk, NY, USA), GraphPad Prism version 9.4.1 (GraphPad Software, Boston, MA, USA), R Studio 2022.07.1+554 and R version 4.2.1 (R Foundation, Vienna, Austria). Categorical variables were analysed by means of Fisher’s Exact or Chi-Square Test. Normal distribution of continuous data was assessed by means of Shapiro-Wilk Test. For comparisons between two groups, Independent Samples T Test was used for normally distributed data, and Mann-Whitney U Test for non-normally distributed data. If more than two groups were compared at a time and data followed a non-normal distribution, overall differences were detected with Kruskal-Wallis Test. Post hoc analysis, if applicable, was conducted by means of Mann-Whitney U Test with Bonferroni correction for multiple comparisons. For comparisons of secretoneurin measurements between very preterm infants and the term-born reference population, 2:1 propensity score matching based on sex and birth weight standard deviation scores (calculated with Fenton percentiles [23]) was used. Associations between two variables were assessed by means of Kendall’s tau-b (τb) correlation coefficient. Linear regression analysis was used to test if secretoneurin concentrations measured at different time points in the neonatal period were predictive of neurodevelopmental outcome at 2 years of age as indicated by Bayley-III composite scores. Significance level for all analyses was set at p<0.05.

## Results

### Study population

**Fig 1:**
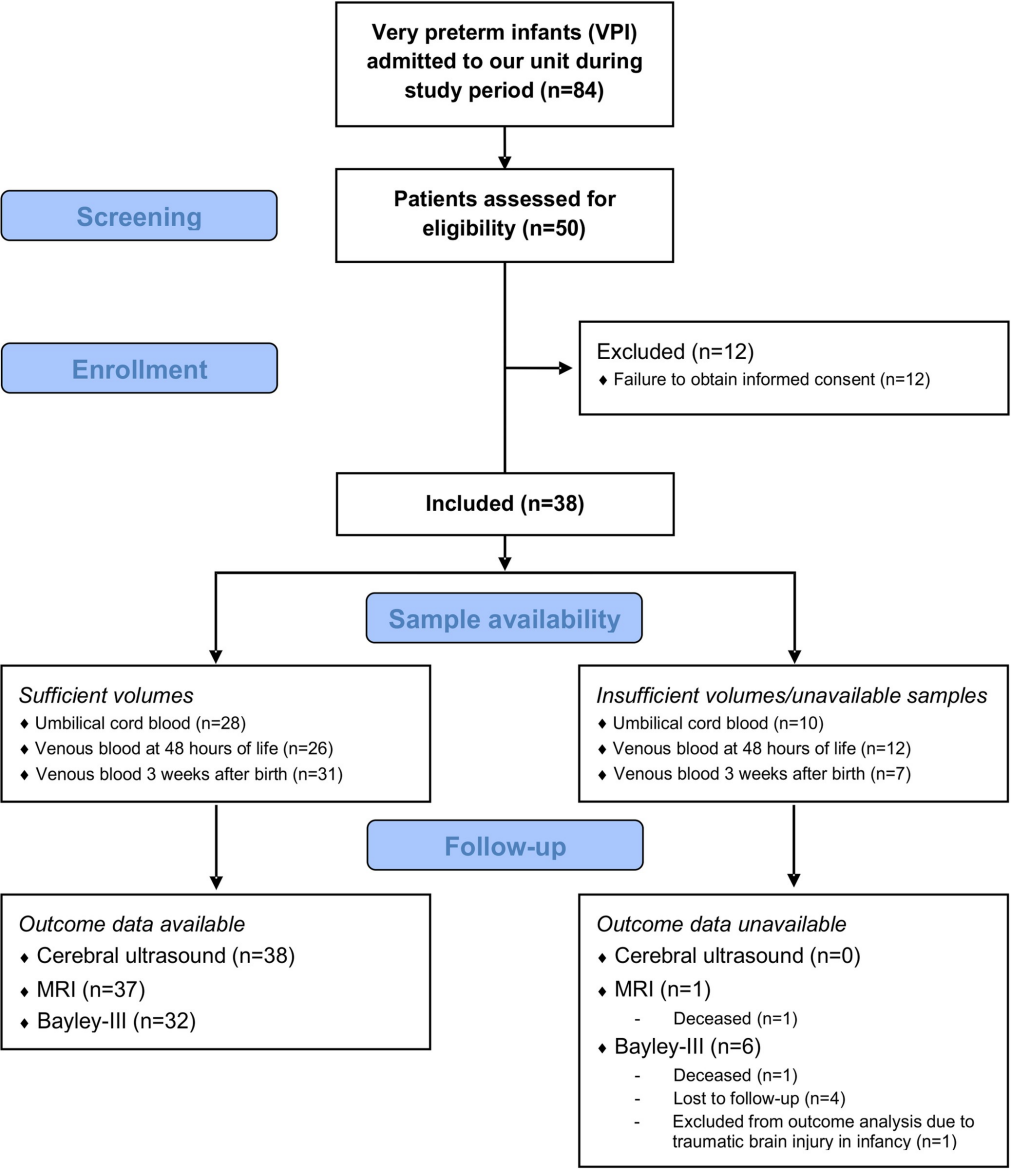
Flow diagram. Overview of all very preterm infants (VPI) admitted to our unit during the study period, patients assessed for eligibility and subjects enrolled in the study. Data availability with regard to secretoneurin measurements and outcome assessments is provided for all VPI included in the study. Data on basic perinatal characteristics were available for all included subjects. *VPI*, very preterm infants.

A total number of 84 very preterm infants were admitted to our unit during the study period, 50 of which were assessed for eligibility for the study. 38 preterm infants were included in the study; 12 were excluded due to failure to obtain informed consent. A flow diagram of the inclusion process and available outcome data can be found in Fig 1. Characteristics of subjects included in the study and all very preterm infants admitted to our unit during the study period are summarized in Table 1.

**Table 1:** Characteristics of included subjects of the study cohort and all very preterm infants (VPI) admitted to our unit during the study period.

Variable			Included subjects (n = 38)	All admitted VPI (n = 84)	p-value
Male, n (%)			18 (47.4)	48 (57.1)	0.334
Birth weight, mean ± SD [g]			1298 ± 372	1255 ± 380	0.566
Gestational age, median (Q1; Q3) [weeks]			29.7 (28.1; 31.4)	29.6 (27.8; 31.1)	0.644
Antenatal corticosteroids administered, n (%)			34 (89.5)	78 (92.9)	0.500
Magnesium sulfate for neuroprotection no/yes/unknown, n (%)			20 (52.6)/16 (42.1)/2 (5.3)	43 (51.2)/39 (46.4)/2 (2.4)	0.676
Type of gestation: multiples, n (%)			16 (42.1)	31 (36.9)	0.688
Mode of delivery: caesarean section, n (%)			37 (97.4)	82 (97.6)	>0.999
Preterm rupture of membranes, n (%)			8 (21.1)	26 (31.0)	0.278
Maternal gravidity, median (Q1; Q3)			2 (1; 3)	1 (1; 3)	0.943
Maternal parity, median (Q1; Q3)			1 (1; 2)	1 (1; 2)	0.579
Apgar 1 minute, median (Q1; Q3)			7 (6; 8)	7 (6; 8)	0.488
Apgar 5 minutes, median (Q1; Q3)			8 (8; 9)	8 (8; 9)	0.455
Apgar 10 minutes, median (Q1; Q3)			9 (9; 9)	9 (9; 9)	0.434
Umbilical cord arterial pH, median (Q1; Q3)			7.34 (7.31; 7.38)	7.34 (7.31; 7.37)	0.414
Chest compressions/adrenaline at birth, n (%)			0 (0.0)	1 (1.2)	>0.999
Catecholamine treatment neonatal period, n (%)			1 (2.6)	6 (7.1)	0.433
Culture-proven early-onset sepsis, n (%)			1 (2.6)	4 (4.8)	>0.999
Culture-proven late-onset sepsis, n (%)			7 (18.4)	11 (13.1)	0.583
Patent ductus arteriosus, n (%)			9 (23.7)	23 (27.4)	0.664
Necrotizing enterocolitis, n (%)^a^			4 (10.5)	4 (4.8)	0.261
Retinopathy of prematurity, n (%)^b^			10 (26.3)	26 (31.0)	0.668
Surgical procedures during birth hospitalization no/yes, n (%)			30 (78.9)/8 (21.1)	66 (78.6)/18 (21.4)	>0.999
Bronchopulmonary dysplasia^c^ treated with systemic corticosteroids, n (%)			5 (13.2)	15 (17.9)	0.603
Cerebral ultrasound pathology, n (%)^d^			5 (13.2)	18 (21.4)	0.327
	GMH-IVH, n (%)		3 (7.9)	10 (11.9)	0.753
		Grade III or IV (PVHI), n (%)	0 (0.0)	3 (3.6)	0.551
	Periventricular leukomalacia, n (%)		1 (2.6)	4 (4.8)	>0.999
	Other, n (%)		2 (5.2)	6 (7.1)	>0.999
MRI: no/mild/severe injury, n (%)^e^			22 (59.5)/12 (32.4)/3 (8.1)	52 (65.0)/23 (28.7)/5 (6.3)	0.880
Head circumference at corrected age of 2 years, mean ± SD [cm]^f^			48.0 ± 1.8	47.9 ± 1.9	0.671
Bayley-III at corrected age of 2 years^g^					
	Cognitive scale, median (Q1; Q3)		100 (90; 114)	100 (90; 115)	0.942
	Language scale, median (Q1; Q3)		97 (76; 121)	97 (81; 117)	0.982
	Motor scale, median (Q1; Q3)		100 (90; 106)	100 (85; 109)	0.901

Data are presented as counts (n) with percentages (%), means ± standard deviations (SD), or medians (quartile 1 (Q1); quartile 3 (Q3)).

^a^As defined by Bell’s criteria.

^b^Classified according to the Committee for the Classification of Retinopathy of Prematurity, unavailable in one included subject (deceased) and four of all VPI admitted to our unit (three deceased, one moved).

^c^Defined as treatment with oxygen >21% for at least 28 days.

^d^Defined as any abnormality observed at any examination time point. Other cerebral ultrasound pathologies include increased echogenicity of unknown aetiology, lenticulostriate vasculopathy and cerebral sinovenous thrombosis.

^e^Graded according to Kidokoro et al. 2014. MRI unavailable in one included subject (deceased) and four of all VPI admitted to our unit (three deceased, one moved).

^f^Head circumference at corrected age of 2 years unavailable in in 6 included subjects and in 15 of all VPI admitted to our unit.

^g^Bayley-III unavailable in 6 included subjects and in 17 of all VPI admitted to our unit.

*Bayley-III*, Bayley Scales of Infant and Toddler Development, third edition; *GMH-IVH*, germinal matrix and intraventricular haemorrhage; *MRI*, magnetic resonance imaging; *PVHI*, periventricular haemorrhagic infarction; *VPI*, very preterm infants.

### Secretoneurin concentrations in very preterm infants

**Fig 2:**
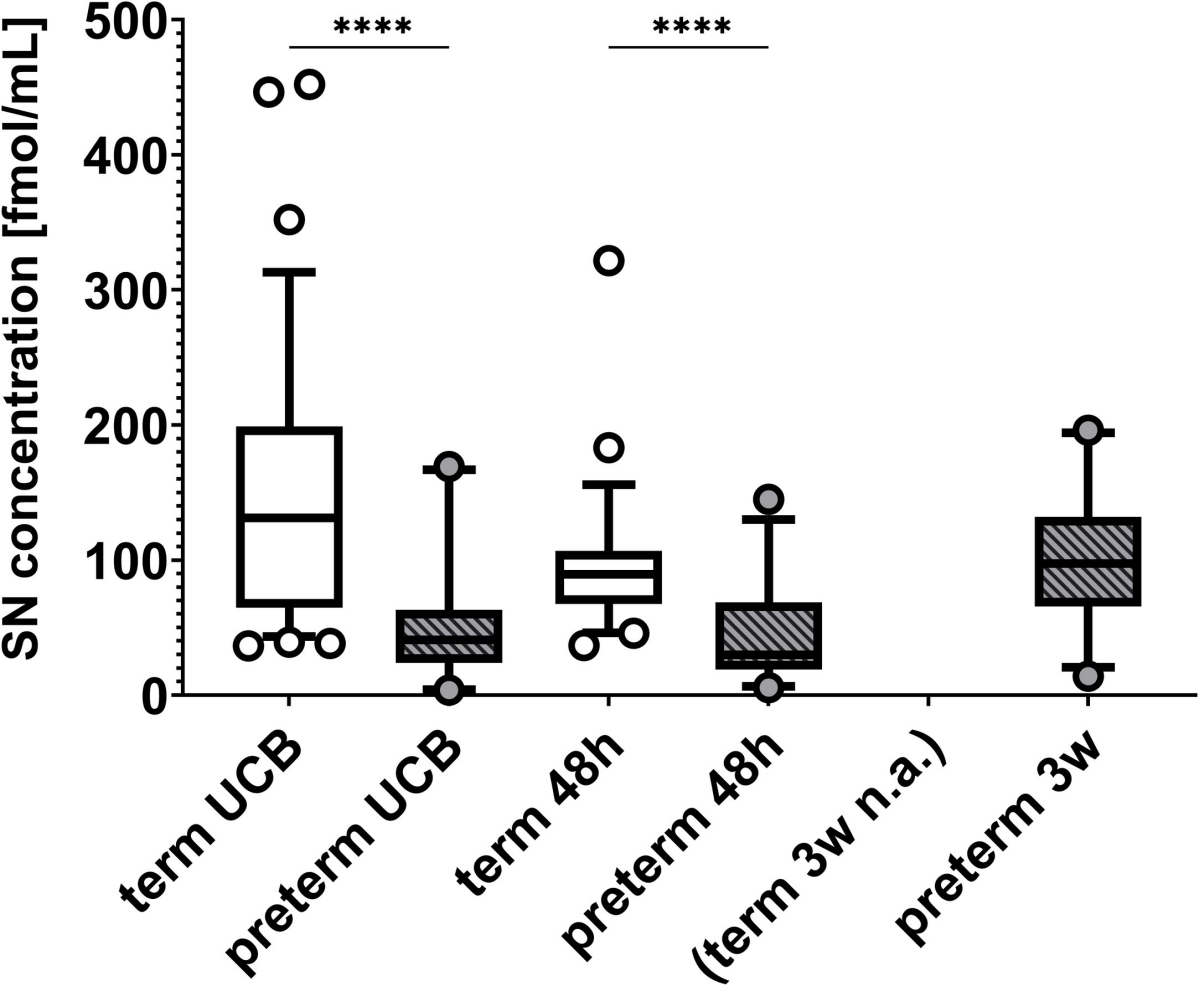
Secretoneurin (SN) levels in healthy term- and preterm-born infants at different time points. SN serum concentrations were determined in umbilical cord blood (UCB), and in patient blood samples obtained at 48 hours (48h) and three weeks (3w) of life. Comparisons were made using 2:1 propensity score matching based on sex and birth weight standard deviation scores (calculated with Fenton 2013 percentiles). Reference values for 3 weeks of life were not available (n.a.). In comparison to term neonates, preterm-born infants had significantly lower SN concentrations in UCB and in blood samples obtained at 48 hours of life. SN concentrations are depicted in fmol/mL. Centre lines in boxes represent medians, box edges mark 1st and 3rd quartiles, and whiskers indicate 5th and 95th percentiles. **** p<0.0001.

**Fig 3:**
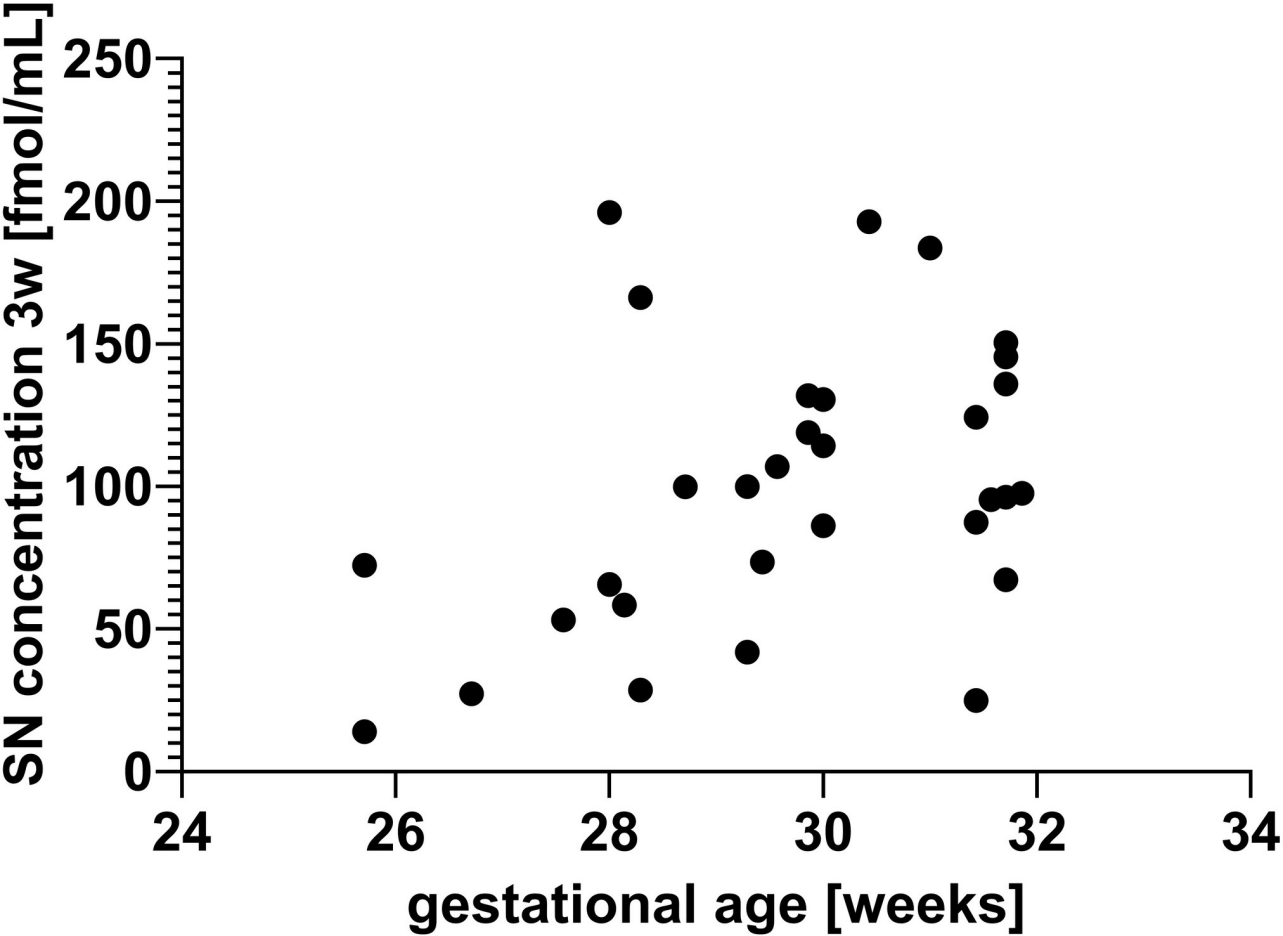
Correlation of secretoneurin (SN) serum concentrations measured in blood samples obtained at 3 weeks (3w) of life with gestational age at birth. Kendall-Tau-b correlation coefficient τb = 0.295, p = 0.022, n = 31. SN concentrations are depicted in fmol/mL. Gestational age is shown in weeks.

Blood samples for secretoneurin serum measurements were obtained from 28 subjects upon delivery (UCB), from 26 subjects at 48 hours, and from 31 subjects at 3 (range 2–4) weeks of life (see also Fig 1). Measurements did not differ between male and female subjects at any time point (all p>0.05, Mann-Whitney U test). In comparison to propensity score-matched term neonates, secretoneurin serum concentrations were significantly lower in preterm-born neonates when determined in UCB (median (quartile 1 (Q1); quartile 3 (Q3)): term 131.2 (64.7; 198.9) fmol/mL, preterm 40.7 (23.8; 63.2) fmol/mL; total n = 104; U = 295, p <0.0001; Mann-Whitney U test) and in patient blood at 48 hours of life median (Q1; Q3): term 89.5 (67.5; 107.0) fmol/mL, preterm 29.9 (18.9; 68.7) fmol/mL; total n = 82; U = 250, p <0.0001; Mann-Whitney U test) (also shown in Fig 2). A multivariate analysis with adjustment for birth mode was not undertaken, as only one preterm study participant was born by vaginal delivery. Exclusion of this participant from the analyses yielded similar results with comparable effect sizes.. In preterm-born infants, secretoneurin concentrations measured in blood samples collected at 3 weeks of life, but not at other time points, were significantly correlated with gestational age at birth (Kendall-Tau-b τb = 0.295, p = 0.022, n = 31) (shown in Fig 3).

### Secretoneurin concentrations and cerebral imaging pathology

**Fig 4:**
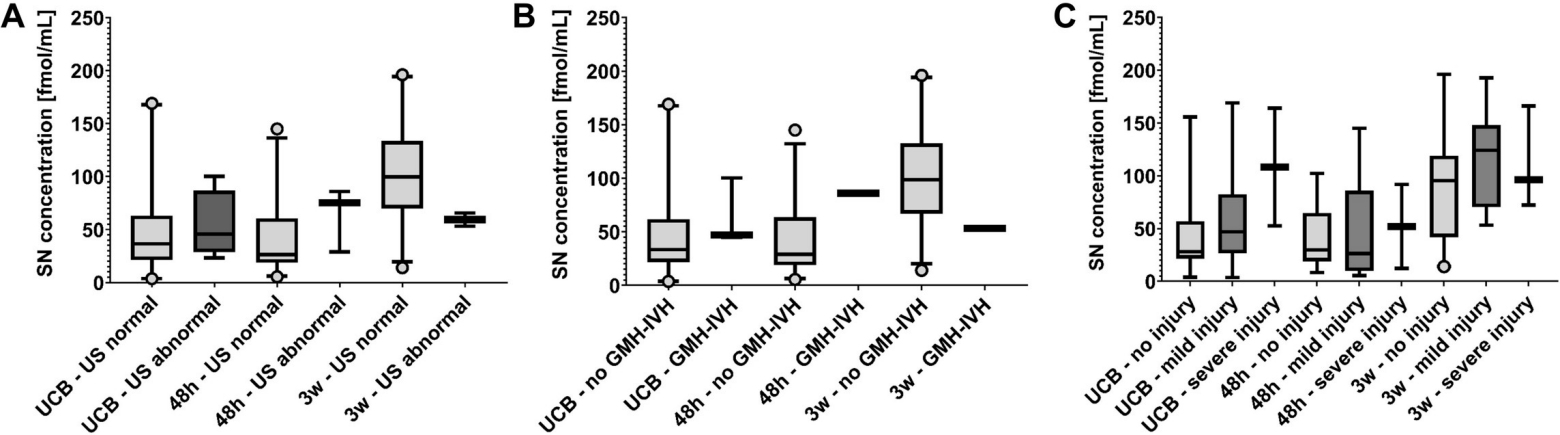
Secretoneurin (SN) levels in preterm infants with cerebral imaging pathology. (A) SN serum concentrations determined in umbilical cord blood (UCB) and in patient blood samples obtained at 48 hours (48h) and three weeks (3w) of life did not significantly differ between preterm infants with a diagnosis of cerebral ultrasound (US) pathology (“US abnormal”) and those without (“US normal”). Number of samples analysed per group and time point: UCB–US normal n = 24, UCB–US abnormal n = 4, 48h –US normal n = 23, 48h –US abnormal n = 3, 3w –US normal n = 29, 3w –US abnormal n = 2. (B) SN serum concentrations determined in umbilical cord blood (UCB) and in patient blood samples obtained at 48 hours (48h) and three weeks (3w) of life did not significantly differ between preterm infants with an ultrasonographic diagnosis of germinal matrix and intraventricular haemorrhage (GMH-IVH) and those without. Number of samples analysed per group and time point: UCB–no GMH-IVH n = 25, UCB–GMH-IVH n = 3, 48h –no GMH-IVH n = 25, 48h –GMH-IVH n = 1, 3w –no GMH-IVH n = 30, 3w –GMH-IVH n = 1. (C) No overall differences were detected in SN serum concentrations determined in umbilical cord blood (UCB) and in patient blood samples obtained at 48 hours (48h) and three weeks (3w) of life in preterm infants with no, mild or severe brain injury on magnetic resonance imaging according to the Kidokoro classification. Number of samples analysed per group and time point: UCB–no injury n = 17, UCB–mild injury n = 9, UCB–severe injury n = 2, 48h –no injury n = 16, 48h –mild injury n = 7, 48h –severe injury n = 2, 3w –no injury n = 19, 3w –mild injury n = 9, 3w –severe injury n = 3. SN concentrations are depicted in fmol/mL. Centre lines in boxes represent medians, box edges mark 1st and 3rd quartiles, and whiskers indicate 5th and 95th percentiles. In case of very small sample sizes in subgroups (n = 1–3), largest and lowest values are depicted.

Secretoneurin serum concentrations measured in UCB and patient blood drawn at 48 hours of life or 3 weeks of life did not significantly differ between preterm infants with cerebral ultrasound pathology in comparison to those with completely normal ultrasound findings (UCB median (Q1; Q3): normal ultrasound 36.5 (21.2; 63.2) fmol/mL, ultrasound pathology 45.8 (28.7; 86.9) fmol/mL; total n = 28, U = 55, p = 0.681; 48 hours median (Q1; Q3): normal ultrasound 26.4 (18.7; 60.7) fmol/mL, ultrasound pathology 75.3 (28.9; 85.9) fmol/mL; total: n = 26, U = 50, p = 0.242; 3 weeks median (Q1; Q3): normal ultrasound 99.9 (69.8; 133.9) fmol/mL, ultrasound pathology 59.4 (53.2; 65.6) fmol/mL; total: n = 31, U = 11, p = 0.181; Mann-Whitney U test) (shown in Fig 4A). Also regarding GMH-IVH versus none, no significant differences were detected (UCB median (Q1; Q3): no GMH-IVH 33.4 (21.6; 61.9) fmol/mL, GMH-IVH 46.9 (44.7; 100.3) fmol/mL; total n = 28, U = 52, p = 0.314; 48 hours median (Q1; Q3): no GMH-IVH 28.9 (18.8; 63.6) fmol/mL, GMH-IVH 85.9 (85.9; 85.9) fmol/mL; total n = 26, U = 21, p = 0.385; 3 weeks median (Q1; Q3): no GMH-IVH 98.7 (66.8; 132.9) fmol/mL, GMH-IVH 53.2 (53.2; 53.2) fmol/mL; total n = 31, U = 5, p = 0.387; Mann-Whitney U test) (shown in Fig 4B). With regard to MRI pathology, no overall significant differences in secretoneurin serum concentrations were detected between preterm infants with no, mild or severe injury (UCB median (Q1; Q3): no injury 28.0 (21.6; 57.1) fmol/mL, mild injury 46.9 (26.7; 82.6) fmol/mL, severe injury 108.3 (52.6; 164.0) fmol/mL; total n = 28, H_(2)_ = 3.612, p = 0.164; 48 hours median (Q1; Q3): no injury 29.9 (19.0; 64.8) fmol/mL, mild injury 26.4 (10.0; 85.9) fmol/mL, severe injury 52.1 (12.3; 91.9) fmol/mL; total n = 25, H_(2)_ = 0.000, p>0.999; 3 weeks median (Q1; Q3): no injury 95.3 (41.9; 118.9) fmol/mL, mild injury 124.3 (70.4; 147.9) fmol/mL, severe injury 96.2 (72.4; 166.2) fmol/mL; total n = 31, H_(2)_ = 1.848, p = 0.397; Kruskall-Wallis test) (shown in Fig 4C).

### Secretoneurin concentrations and unfavourable outcomes

**Fig 5:**
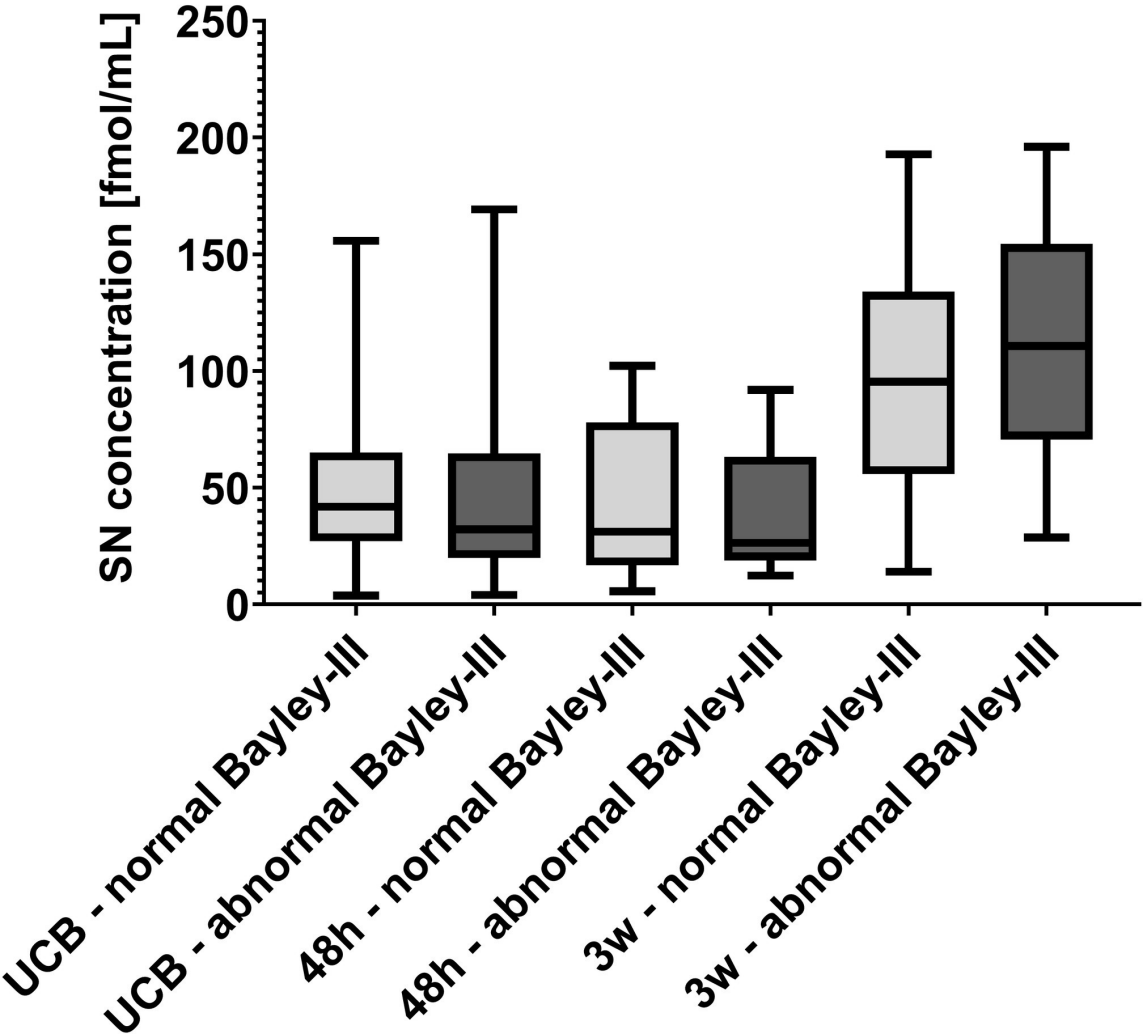
Secretoneurin (SN) levels in preterm infants with unfavourable outcome at the age of 2 years corrected age. No significant differences were detected in SN serum concentrations determined in umbilical cord blood (UCB) and in patient blood samples obtained at 48 hours (48h) and three weeks (3w) of life in preterm infants with abnormal neurodevelopmental outcome defined as a score of <85 in any of the three major domains of the Bayley Scales of Infant and Toddler Development, third edition (Bayley-III). Number of samples analysed per group and time point: UCB–normal Bayley-III n = 15, UCB–abnormal Bayley-III n = 11, 48h –normal Bayley-III n = 14, 48h –abnormal Bayley-III n = 9, 3w –normal Bayley-III n = 17, 3w –abnormal Bayley-III n = 10. SN concentrations are depicted in fmol/mL. Centre lines in boxes represent medians, box edges mark 1st and 3rd quartiles, and whiskers indicate 5th and 95th percentiles.

With regard to outcome assessments, one study participant died from advanced necrotizing enterocolitis (Bell grade IIIB) at 20 days of life and data was thus censored for this participant. At 52 weeks postmenstrual age, fidgety movements were present in all remaining study participants and general movements were thus categorized as normal in all evaluated subjects. With regard to neurodevelopmental outcome at a corrected age of 2 years, one participant was excluded from the analysis due to traumatic brain injury at 5 months at age. Further details on available outcome data can be found in Fig 1. No statistically significant differences were detected in secretoneurin serum concentrations in UCB or patient blood drawn at 48 hours or 3 weeks of life between children with abnormalities detected by Bayley-III examination and those with completely normal findings (UCB median (Q1; Q3): normal Bayley-III 41.8 (27.1; 65.0) fmol/mL, abnormal Bayley-III 32.1 (19.9; 64.6) fmol/mL; total n = 26, U = 76, p = 0.760; 48 hours median (Q1; Q3): normal Bayley-III 31.1 (16.8; 77.9) fmol/mL, abnormal Bayley-III 26.4 (18.8; 63.2) fmol/mL; total n = 23, U = 57, p = 0.734; 3 weeks median (Q1; Q3): normal Bayley-III 95.3 (55.8; 133.9) fmol/mL, abnormal Bayley-III 110.6 (70.7; 154.3) fmol/mL; total: n = 27, U = 67, p = 0.386; Mann-Whitney U test) (shown in Fig 5).

**Fig 6:**
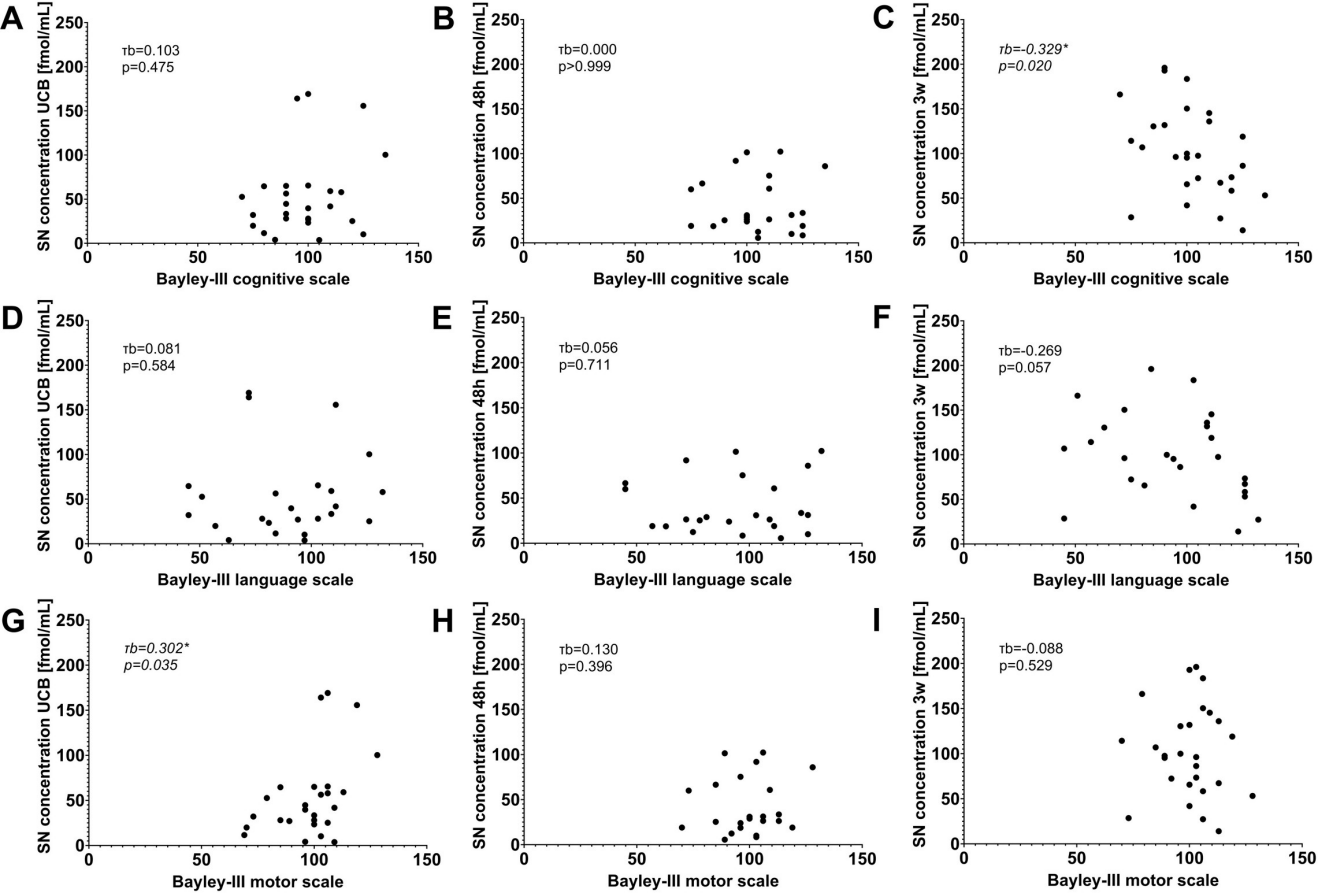
Correlation of secretoneurin (SN) serum concentrations with Bayley Scales of Infant and Toddler Development, third edition (Bayley-III) assessed at a corrected age of 2 years. Composite scores for the cognitive scale (A-C), the language scale (D-F), and the motor scale (G-I) are presented. SN concentrations were measured in umbilical cord blood (UCB) (A, D, G) and in blood samples collected at 48 hours (48h) (B, E, H) and 3 weeks (3w) of life (C, F, I). Associations between two variables were assessed by means of Kendall’s tau-b (τb) correlation coefficient. Significance level was set at p<0.05. Significant correlations were detected for SN concentrations measured in UCB and Bayley-III motor scale scores as well as SN concentrations determined at 3w and Bayley-III cognitive scale scores. SN concentrations are depicted in fmol/mL.

Secretoneurin serum concentrations determined in UCB were significantly correlated with Bayley-III motor scale scores (Kendall-Tau-b τb = 0.302, p = 0.035, n = 26), but not with other scales. Secretoneurin concentrations measured in blood samples collected at 48 hours of life did not show significant correlations with any of the major Bayley-III scales. Secretoneurin concentrations measured in blood samples collected at 3 weeks of life were inversely correlated with Bayley-III cognitive scale scores (Kendall-Tau-b τb = -0.329, p = 0.020, n = 27). Correlations of all secretoneurin measurements and Bayley-III composite scores are shown in Fig 6.

Linear regression analysis was used to test if secretoneurin concentrations measured at various time points in the neonatal period significantly predicted Bayley-III composite scores at a corrected age of 2 years. In a simple linear regression model, secretoneurin serum concentrations determined in UCB significantly predicted Bayley-III motor scale scores (fitted regression equation: motor scale = 91.7+0.129 (secretoneurin concentration UCB); overall regression: R² = 0.192, F_(1,24)_ = 5.686, p = 0.025; predictor secretoneurin concentration UCB: ß = 0.129, p = 0.025). Secretoneurin concentration in UCB remained a significant predictor of Bayley-III motor scale scores after addition of gestational age to the regression analysis (fitted regression equation: motor scale score = 88.3+0.128 (secretoneurin concentration UCB) + 0.119 (gestational age); overall regression: R² = 0.192, F_(2,23)_ = 2.729, p = 0.086; predictor secretoneurin concentration UCB: ß = 0.128, p = 0.042; predictor gestational age: ß = 0.119, p = 0.932). Secretoneurin concentrations measured in blood samples collected at 48 hours of life were not predictive of any Bayley-III scales (all p≥0.05). Secretoneurin concentrations measured in blood samples collected at 3 weeks of life significantly predicted Bayley-III cognitive scale scores (fitted regression equation: cognitive scale score = 114.9–0.136 (secretoneurin concentration 3 weeks); overall regression: R² = 0.175, F_(1,25)_ = 5.311, p = 0.030; predictor secretoneurin concentration 3 weeks: ß = -0.136, p = 0.030). Secretoneurin concentration measured in blood samples collected at 3 weeks of life remained a significant predictor of Bayley-III cognitive scale scores after addition of gestational age to the regression analysis (fitted regression equation: cognitive scale score = 61.2–0.172 (secretoneurin concentration 3 weeks) + 1.953 (gestational age); overall regression: R² = 0.218, F_(2,24)_ = 3.337, p = 0.053; predictor secretoneurin concentration 3 weeks: ß = -0.172, p = 0.016; predictor gestational age: ß = 1.953, p = 0.266).

## Discussion

The main findings of our pilot study can be summarized as follows: 1) Secretoneurin concentrations in very preterm infants are lower than in full-term neonates both in umbilical cord blood and in blood collected at 48 hours of life. 2) When measured at 3 weeks of life, secretoneurin concentrations in very preterm infants correlate with gestational age at birth. 3) Secretoneurin concentrations do not differ between very preterm infants with an imaging-based diagnosis of brain injury and those without. 4) When measured in umbilical cord blood and at 3 weeks of life, secretoneurin concentrations correlate with and are predictive of Bayley-III motor and cognitive scale scores at 2 years of age.

### Secretoneurin and prematurity

In our very preterm cohort, secretoneurin concentrations were significantly lower than in a propensity score-matched term-born reference population in umbilical cord blood and at 48 hours of life. We observed an increase 3 weeks after birth with no reference data available, but concentrations approached those measured in term-born neonates in the first 48 hours of life [13]. At that time point, secretoneurin concentrations also correlated with gestational age at birth, indicating possible maturational effects. These findings are in accordance with a previous report by Leitner and colleagues describing the ontogenic development of secretoneurin in rodent brain tissue. They detected secretoneurin-immunopositive cells from embryonic day 13 on, with a strong increase from day 14 to 18 in several brain areas [24]. The further course of secretoneurin concentrations in very preterm infants is currently unclear, as measurements up to term-equivalent age were not undertaken in the present study due to its focus on preterm brain injury, which occurs mainly in the first month of life. The temporal and maturational dynamics of secretoneurin synthesis merit further investigation in the future.

### Secretoneurin and cerebral imaging pathology

Based on previous literature reporting increases in secretoneurin concentrations in association with adult brain injury [11, 12], and particularly in full-term neonates suffering from perinatal asphyxia with hypoxic-ischaemic brain injury [13], we hypothesized that secretoneurin might also be increased in very preterm infants with cerebral imaging pathology, but detected no significant differences between infants with apparent brain injury and those without on ultrasound or magnetic resonance imaging. These results might seem discordant at first, but may actually reflect the different pathophysiology of brain injury in preterm and term neonates. Hypoxic-ischaemic encephalopathy occurring in perinatal asphyxia is caused by a sudden lack of oxygen supply to the brain shortly before, during or after birth due to an abrupt event. Preterm brain injury, on the other hand, is associated with axonal/neuronal damage, white matter abnormalities and germinal matrix/intraventricular haemorrhage. Its aetiology involves intricate interactions among apparent parenchymal damage and alterations on a cellular level, which lead to more subtle changes (6). It evolves over time, is often aggravated by multiple hits, and represents a long-term disruption of physiological brain development [6, 25], which can lead to an unfavourable neurodevelopmental outcome even in the absence of cerebral imaging pathology [26].

### Secretoneurin and neurodevelopmental outcome

Even though we found no differences in secretoneurin concentrations between very preterm infants with an imaging-based diagnosis of brain injury and those without, secretoneurin concentrations to a certain extent predicted neurodevelopmental outcome: Firstly, secretoneurin serum concentrations determined in UCB positively correlated with and significantly predicted Bayley-III motor scale scores, with lower concentrations being associated with lower scores. Bearing in mind the neuron-conserving, anti-apoptotic, neurogenic and angiogenic properties of secretoneurin [12], we can speculate that this reflects a “depleted pool” of potentially neuroprotective secretoneurin in early injury phases. This is corroborated by findings in a mouse model of preterm brain injury, where animals subjected to excitotoxic injury had significantly lower secretoneurin concentrations 6 hours after insult in comparison to healthy controls [27]. In contrast to this, secretoneurin concentrations measured in blood samples collected at 3 weeks of life inversely correlated with and significantly predicted Bayley-III cognitive scale scores, with higher concentrations being associated with lower scores. Elevated secretoneurin concentrations later in life may be indicative of repeated insults. In their clinical course, very preterm infants frequently experience hypoxaemic episodes [28]. As secretoneurin expression is induced by cellular hypoxia [14], these episodes are a potential stimulus for its upregulation. In addition, secretoneurin is known to be involved in inflammatory processes [14], which play a crucial role in later phases of preterm brain injury and can be associated with unfavourable outcomes [15].

### Secretoneurin and comorbid conditions

In adult populations, secretoneurin levels have been shown to provide prognostic information in critically ill patients with severe infections, especially in cases with haemodynamic instability [29, 30], and in patients with cardiovascular disease [31, 32]. Secretoneurin is also known to be elevated after cardiopulmonary resuscitation [11]. Data on extra-cerebral comorbidities impacting on secretoneurin levels in very preterm infants is lacking to date.

We cannot rule out an effect of cardiovascular compromise on secretoneurin concentrations in very preterm infants, as the number of patients requiring chest compressions/adrenaline at birth or catecholamine treatment in the neonatal period in our study cohort was very low (n = 0 and n = 1, respectively; see also Table 1). With regard to severe infections, we did not observe significant differences in secretoneurin concentrations measured at all three time points between very preterm infants with a diagnosis of perinatal infection/inflammation (necrotizing enterocolitis and/or sepsis) and those without (for details see S1 Fig). Both aspects merit investigation in future studies with larger cohorts.

### Limitations and strengths of the study

Our study’s major limitations are the limited sample size and the low number of preterm-born infants with cerebral imaging pathology and abnormal Bayley-III outcomes, making interpretation of our results challenging. However, our study cohort is representative of all very preterm infants admitted to our unit during the study period and it did not differ from the very preterm population at our NICU in relevant perinatal characteristics, complications of preterm birth or neurodevelopmental outcome (see also Table 1). Our study’s major strengths are the comprehensive assessments and standardized follow-up examinations of the highly vulnerable very preterm population.

## Conclusions

In our observational pilot study, we determined secretoneurin serum concentrations in very preterm infants from birth to 3 weeks of life. In comparison to term-born neonates, secretoneurin concentrations were significantly lower in preterm infants in umbilical cord blood and at 48 hours of life. When measured at 3 weeks of life, secretoneurin concentrations correlated with gestational age at birth. We found no differences in secretoneurin serum concentrations between preterm infants with and without an imaging-based diagnosis of brain injury, but secretoneurin concentrations to a certain extent predicted neurodevelopmental outcome at 2 years of age. Secretoneurin probably underlies a substantial variation in preterm-born neonates and further studies are required to confirm the findings of our pilot study before it can be put to larger-scale clinical use in identifying children at risk for adverse neurodevelopmental outcomes.

## Supporting information

S1 Fig Secretoneurin (SN) levels in very preterm infants with and without perinatal infection/inflammation. No significant differences were detected in SN serum concentrations determined in umbilical cord blood (UCB) and in patient blood samples obtained at 48 hours (48h) and three weeks (3w) of life in very preterm infants with a diagnosis of perinatal infection/inflammation (inf/infl; including necrotizing enterocolitis and/or sepsis) and those without (no inf/infl). Number of samples analysed per group and time point: UCB–no inf/infl n = 22, UCB–inf/infl n = 6, 48h –no inf/infl n = 20, 48h –inf/infl n = 6, 3w –no inf/infl n = 24, 3w –inf/infl n = 7. SN concentrations are depicted in fmol/mL. Centre lines in boxes represent medians, box edges mark 1st and 3rd quartiles, and whiskers indicate 5th and 95th percentiles.
(TIF)

S2 Fig Correlation of secretoneurin (SN) serum concentrations with head circumference measured at a corrected age of 2 years (2y). No significant correlations were detected between SN concentrations measured in umbilical cord blood (UCB) (A) and in blood samples collected at 48 hours (48h) (B) and 3 weeks (3w) of life (C) and head circumference at 2y. Associations between two variables were assessed by means of Kendall’s tau-b (τb) correlation coefficient. Significance level was set at p<0.05. SN concentrations are depicted in fmol/mL.
(TIF)

S1 File Supplementary materials and methods. Collection of basic perinatal and neonatal data as well as neurodevelopmental outcome assessment.
(DOCX)
