# Supplementary figures and images for: Investigation of secretoneurin as a potential biomarker of brain injury in very preterm infants: A pilot study
Source: PLoS One. 2023 Apr 6;18(4):e0284096. doi: 10.1371/journal.pone.0284096 (PMC10079118; doi:10.1371/journal.pone.0284096)

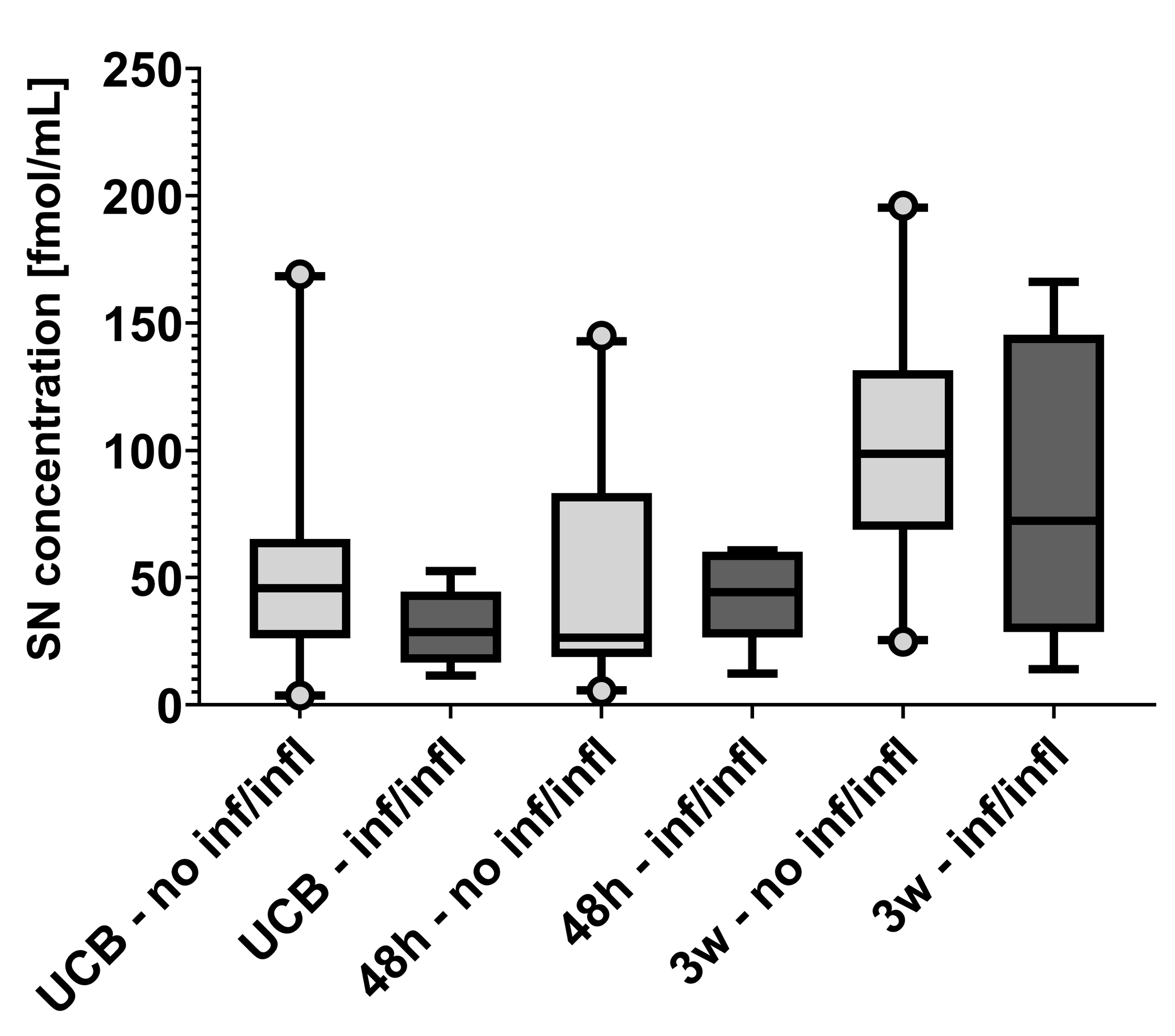

Supplement: S1 Fig — No significant differences were detected in SN serum concentrations determined in umbilical cord blood (UCB) and in patient blood samples obtained at 48 hours (48h) and three weeks (3w) of life in very preterm infants with a diagnosis of perinatal infection/inflammation (inf/infl; including necrotizing enterocolitis and/or sepsis) and those without (no inf/infl). Number of samples analysed per group and time point: UCB–no inf/infl n = 22, UCB–inf/infl n = 6, 48h –no inf/infl n = 20, 48h –inf/infl n = 6, 3w –no inf/infl n = 24, 3w –inf/infl n = 7. SN concentrations are depicted in fmol/mL. Centre lines in boxes represent medians, box edges mark 1st and 3rd quartiles, and whiskers indicate 5th and 95th percentiles. (TIF) [file pone.0284096.s001.tif]

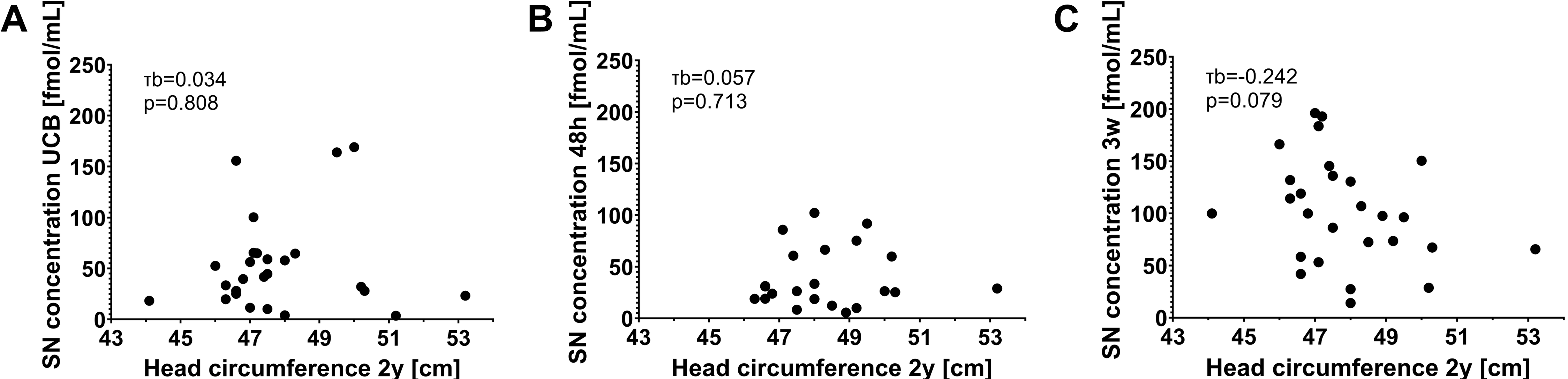

Supplement: S2 Fig — No significant correlations were detected between SN concentrations measured in umbilical cord blood (UCB) (A) and in blood samples collected at 48 hours (48h) (B) and 3 weeks (3w) of life (C) and head circumference at 2y. Associations between two variables were assessed by means of Kendall’s tau-b (τb) correlation coefficient. Significance level was set at p<0.05. SN concentrations are depicted in fmol/mL. (TIF) [file pone.0284096.s002.tif]
