# Supplementary material for: Investigation of secretoneurin as a potential biomarker of brain injury in very preterm infants: A pilot study
Source: PLoS One. 2023 Apr 6;18(4):e0284096. doi: 10.1371/journal.pone.0284096 (PMC10079118; doi:10.1371/journal.pone.0284096)
Supplement: S1 File — Collection of basic perinatal and neonatal data as well as neurodevelopmental outcome assessment. (DOCX) [file pone.0284096.s003.docx]

# Supplementary material

## Supplementary materials and methods

### Collection of basic perinatal and neonatal data

Basic perinatal and neonatal data were collected during the hospital stay as described previously [1]. In brief, collected data included gender, birth weight, gestational age (full weeks of gestation), use of antenatal steroids, magnesium sulfate for neuroprotection, type of gestation (multiple pregnancies), mode of delivery, timing of rupture of membranes, maternal gravidity and parity, Apgar scores, umbilical cord arterial pH, resuscitative measures at birth, and relevant complications of preterm birth such as requirement for catecholamine treatment in the neonatal period, culture-proven early-onset (within first 72 hours after birth) and late-onset (at ≥72 hours of age) sepsis, patent ductus arteriosus, necrotizing enterocolitis, retinopathy of prematurity, surgical procedures during birth hospitalization, and bronchopulmonary dysplasia requiring systemic corticosteroid treatment. Necrotizing enterocolitis was defined according to Bell’s criteria [2], retinopathy of prematurity according to the international classification of the Committee for the Classification of Retinopathy of Prematurity [3]. Bronchopulmonary dysplasia was defined as treatment with oxygen >21% for at least 28 days [4]. Perinatal data including neuroimaging results and neurodevelopmental outcome assessments of included subjects of the study cohort and all very preterm infants admitted to our unit during the study period are presented in Table 1.

### Neurodevelopmental outcome assessment

At a corrected age of two years, all former very preterm infants were invited to a routine visit at our preterm follow-up clinic. Head circumference was measured with a flexible non-stretchable measuring tape. Neurodevelopmental outcome was assessed by Bayley Scales of Infant and Toddler Development, third edition (Bayley-III) [5]. The Bayley-III produces three composite scores: a cognitive scale (range 55-145), which assesses sensorimotor development, exploration, manipulation, object relatedness, concept of formation, memory and simple problem solving; a language scale (range 45–155), which evaluates receptive and expressive communication; and a motor scale (range 45–155), which assesses fine and gross motor function. Each scale is age-standardized with a mean of 100 (standard deviation (SD): 15), with a score of less than 85 (>1 SD below the mean) indicating mild delay and a score of less than 70 (>2 SDs below the mean) indicating severe impairment. Abnormal neurodevelopmental outcome was defined as a score of <85 comprising delay (score 70-84) and impairment (score <70) in any of the three domains mentioned above. All tests were performed by experienced psychologists.

## Supplementary references

1. Neubauer, V., Griesmaier, E., Pehbock-Walser, N., Pupp-Peglow, U. & Kiechl-Kohlendorfer, U. Poor Postnatal Head Growth in Very Preterm Infants Is Associated with Impaired Neurodevelopment Outcome. *Acta Paediatr* **102**, 883-888 (2013).

2. Bell, M. J. et al. Neonatal Necrotizing Enterocolitis. Therapeutic Decisions Based Upon Clinical Staging. *Ann Surg* **187**, 1-7 (1978).

3. An International Classification of Retinopathy of Prematurity. The Committee for the Classification of Retinopathy of Prematurity. *Arch Ophthalmol* **102**, 1130-1134 (1984).

4. Jobe, A. H. & Bancalari, E. Bronchopulmonary Dysplasia. *Am J Respir Crit Care Med* **163**, 1723-1729 (2001).

5. Albers CA, G. A. Bayley Scales of Infant and Toddler Development, Third Edition. *Journal of Psychoeducational Assessment* **Jun;25(2):180-90.** (2007).
